# 2545. Impact of Early Dose Adjustment of Beta-Lactams in Patient with Septic Shock

**DOI:** 10.1093/ofid/ofad500.2162

**Published:** 2023-11-27

**Authors:** John C Tawwater, David T Adams, Satwinder S Kaur, Nayle Ibragimova

**Affiliations:** Texas Health Harris Methodist Hospital Fort Worth, Fort Worth, Texas; Texas Health Harris Methodist Hospital Fort Worth, Fort Worth, Texas; Texas Health Harris Methodist Hospital Fort Worth, Fort Worth, Texas; University of Oklahoma Health Sciences Center, Harker Heights, Texas

## Abstract

**Background:**

Effective antimicrobial dosing is crucial for survival in patient with septic shock. However, attaining therapeutic antimicrobial serum concentration is often problematic due to pathophysiological changes associated with critical illness. Antimicrobial concentrations can be decreased due to increased volume of distribution and augmented renal clearance. In addition, antibiotic doses are frequently adjusted per local renal dosing protocols based on creatinine clearance, resulting in suboptimal antibiotic exposures. The purpose of this study is to determine if unadjusted dosing of cefepime (CEF) and meropenem (MER) during the first 48 hours of septic shock improves clinical outcomes.

**Methods:**

This is a retrospective observational cohort study comparing renally dose adjusted CEF and MER per institutional renal dosing protocol (pre-group, PRE) and unadjusted, full-dose CEF and MER (post-group, POST) (Figure 1). Adults aged 18-89 years with septic shock, initially treated with CEF or MER for at least 48 hours, and presenting with renal impairment are included. Patients receiving renal replacement therapies are excluded. The primary outcome is vasopressor-free days (VFD). Secondary outcomes are composite outcome of in-hospital mortality and hospice disposition, hospital length of stay (LOS), intensive care unit (ICU) LOS, and systemic inflammatory response syndrome (SIRS)-free days.

**Figure 1.**
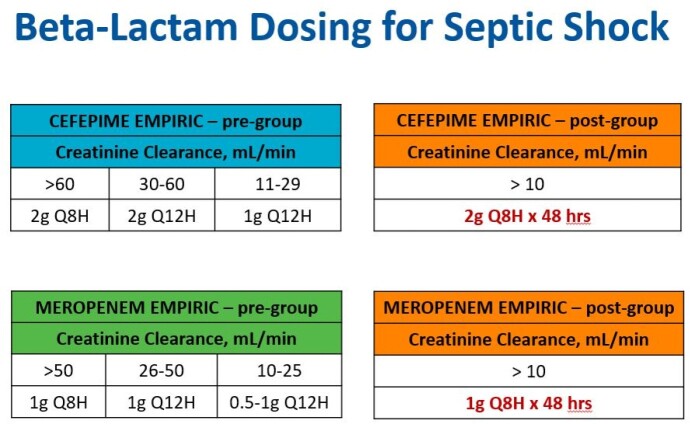
Beta-lactam Dosing for Septic Shock - PRE and POST.

**Results:**

Baseline demographic characteristics were similar between the groups with the exception of receipt of stress-dose steroids, which was significantly higher in PRE (p=0.019). The primary outcome of VFD was not significantly different between the groups, with a median of 9.33 days in PRE (n=40) and 9.54 days in POST (n=32) (Figure 2). The hospital LOS (PRE 12.5 days, POST 5.25 days, p=0.121), ICU LOS (PRE 5.5 days, POST 5 days, p=0.15), mortality, and SIRS-free days were not significantly different between the groups (Figure 3).

**Figure 2.**
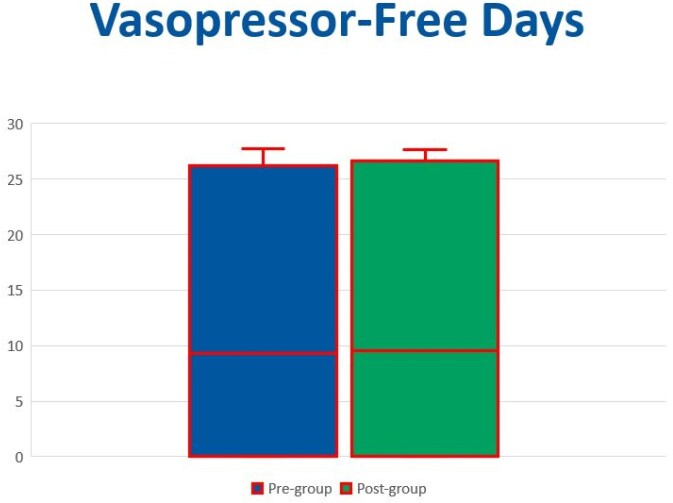
Primary outcome - vasopressor-free days.

**Figure 3.**
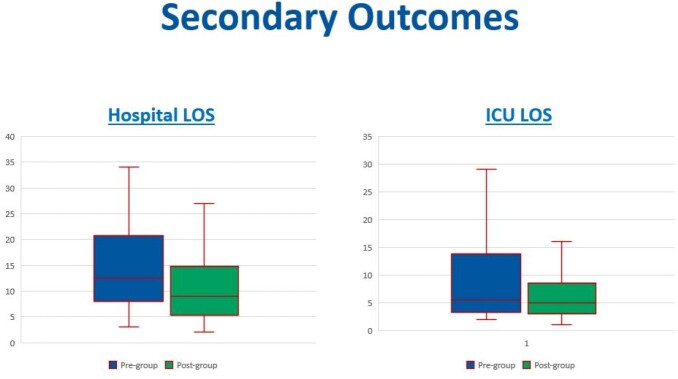
Secondary outcomes - hospital LOS and ICU LOS.

**Conclusion:**

Unadjusted beta-lactam dosing did not increase VFD, although the significantly higher use of stress-dose steroids in PRE could confound the result. There was no differences in secondary efficacy outcomes; however, an overall trend for shorter hospital LOS and ICU LOS in POST was observed.

**Disclosures:**

**All Authors**: No reported disclosures

